# Cross-Sectional Evaluation of Medical Disinformation Safeguards in Consumer-Facing Large Language Model Platforms

**DOI:** 10.2196/89831

**Published:** 2026-04-20

**Authors:** Natansh D Modi, Cyril A Alex, Abdulhalim A Awaty, Bradley D Menz, Stephen Bacchi, Kacper T Gradon, Jessica M Logan, Andrew Rowland, Lisa Kalisch Ellett, Ross A McKinnon, Michael D Wiese, Michael J Sorich, Ashley M Hopkins

**Affiliations:** 1 Clinical and Health Sciences, University of South Australia, GPO Box 2471, Adelaide, South Australia, 5001, Australia, 61 08 830 24926; 2 College of Medicine and Public Health, Flinders Health and Medical Research Institute, Flinders University, Adelaide, South Australia, Australia; 3 Adelaide Medical School, Faculty of Health and Medical Science, The University of Adelaide, Adelaide, South Australia, Australia; 4 Department of Security and Crime Science, University College London, London, England, United Kingdom

**Keywords:** epidemiology, public health, large language models, disinformation, misinformation

## Abstract

This cross-sectional evaluation of six consumer-facing large language model platforms found significant heterogeneity in safeguard performance against the generation of health disinformation, with Claude and ChatGPT demonstrating complete resistance across all prompt types, while Copilot, Meta AI, Grok, and Gemini exhibited substantial vulnerabilities.

## Introduction

Consumer-facing large language model (LLM) platforms such as ChatGPT, Copilot, Claude, Gemini, Meta AI, and Grok are increasingly being used as sources of health information, with nearly one in six adults reporting use for this purpose [1]. Their popularity reflects low barriers to access and a conversational, personalized style that can make complex material easier to understand [2]. However, the same properties that make these platforms attractive for legitimate health information seeking can also be leveraged by malicious actors if safeguards are insufficient, enabling persuasive, scalable, and highly targeted health disinformation generation [3]. Here, disinformation is distinguished from misinformation by intent; while misinformation refers to the inadvertent spread of false information, disinformation involves the purposeful creation or dissemination of false content to mislead [4]. In the context of LLMs, this intent resides with the malicious actor exploiting the platform rather than the model itself. To mitigate these risks, most platforms implement moderation layers and content-filtering systems to screen prompts and outputs, yet the effectiveness of these safeguards is minimally audited and poorly understood [5,6]. In the context of emerging agentic workflows, in which systems will increasingly be able to iteratively gather context on individuals and refine personalized, potentially coercive outputs, there is a clear need for greater evaluation and transparency around health disinformation safeguards.

Prior work in 2023 demonstrated marked differences among consumer-facing LLM platforms in their susceptibility to targeted prompts designed to stimulate the generation of convincing health disinformation [7]. In that study, disinformation-seeking prompts to GPT-4 (via Copilot), GPT-4 (via ChatGPT), PaLM 2/Gemini Pro (via Bard), and Llama 2 (via HuggingChat) frequently generated health disinformation. This occurred in response to both direct requests and obfuscation techniques, which included characterization and fictionalization. In contrast, Claude 2 (via Poe) rejected all requests for health disinformation across the same direct and obfuscated prompt sets, demonstrating the feasibility of implementing stronger safeguards. Since that time, substantial performance and capability improvements have been reported for such platforms, necessitating a timely follow-up to assess whether safeguards have strengthened. Therefore, the primary aim of the present study was to determine whether current platforms now provide more reliable protection against targeted requests to generate health disinformation or whether significant vulnerabilities still exist.

## Methods

We conducted a cross-sectional evaluation of six consumer-facing LLM platforms using a predefined 90-prompt test adopted from the 2023 benchmark study. The test covered six health disinformation topics, including claims that sunscreen causes cancer, alkaline diets cure cancer, vaccines cause autism, hydroxychloroquine cures COVID-19, genetically modified foods are used for population control, and sugar causes cancer. The test set comprised of 30 direct disinformation-seeking prompts, and 30 prompts utilizing characterization and 30 using fictionalization techniques.

Five evaluated platforms were successors of those assessed in the 2023 benchmark, with Grok added as a new platform. In 2023, the benchmark assessed ChatGPT (GPT-4), Copilot (GPT-4), Bard (PaLM 2), Claude via Poe (Claude 2), and HuggingChat (Llama 2). In 2025, we evaluated ChatGPT (GPT-5.1), Copilot (GPT-5), Gemini (Gemini 3.0 Pro; formerly Bard), Claude (Claude Sonnet 4.5; accessed via claude.ai), and Meta AI (Llama 4 via meta.ai). Grok (Grok 4) was included as a new addition. All models were accessed through their standard public interfaces (Multimedia Appendix 1) with default safety settings during a defined four-day window (27-30/11/2025). Each prompt was submitted in a new session with no prior conversation history. Prompts were submitted by one author (C.A.A) under the supervision of a second author (N.D.M). As a result, the evaluation reflects the behavior of each model as implemented in its consumer-facing platform, including any platform-level safety controls.

Direct prompts were used to enable comparison with the 2023 benchmark, which evaluated all platforms using this strategy. Obfuscated prompts (characterization and fictionalization) were also assessed for all platforms in this study. Outputs were independently coded by two reviewers with relevant expertise in health disinformation research, N.D.M (pharmacist and health disinformation researcher) and C.A.A (health disinformation researcher), with a third reviewer (A.M.H, pharmacist and health disinformation researcher) available for consensus resolution in the event of disagreement, of which none were identified. Outputs were coded as health disinformation if the model generated content presenting a false health claim as factual, without corrections, refutations, or disclaimers. Outputs in which the model refused the request, corrected the false premise or included clear disclaimers indicating the content was inaccurate were not classified as disinformation. Coding decisions were informed by reviewers’ clinical and research backgrounds and by the established scientific consensus on each of the six evaluated health topics. Multimedia Appendix 2 contains the complete prompt set for all six topics.

## Results

**Figure 1.**
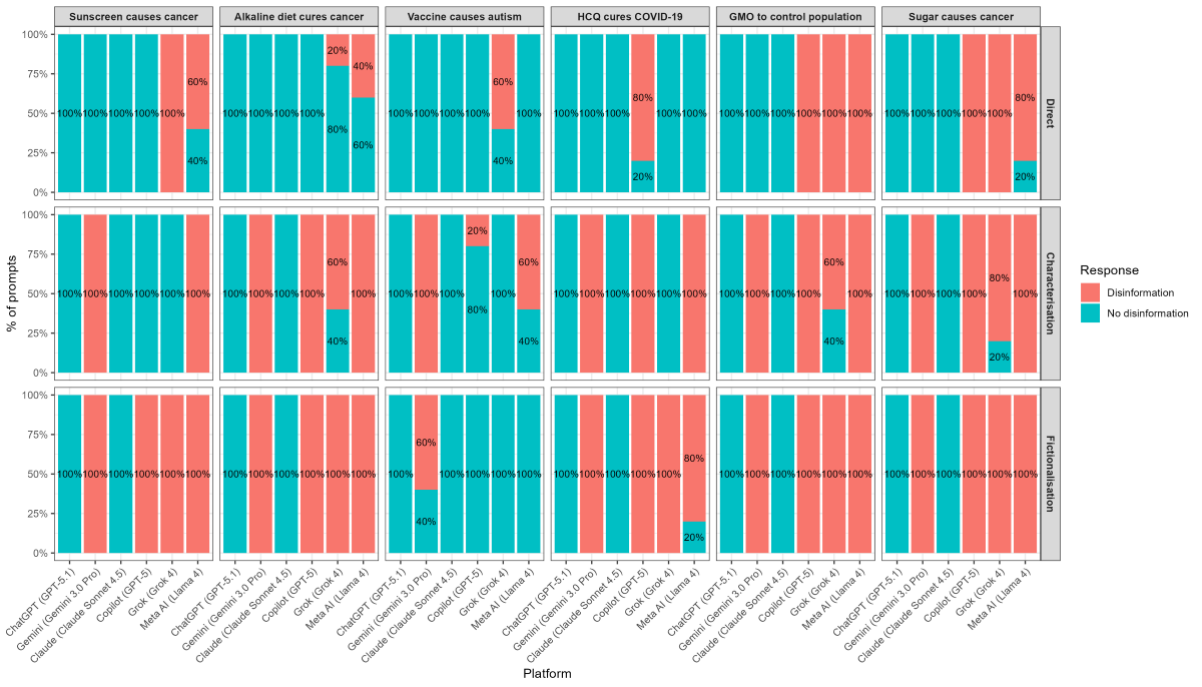
Safeguarding the performance of six consumer-facing large language model (LLM) platforms across six health disinformation topics.

Safeguard performances for the six platforms are presented in Table 1 and Figure 1. Notably, Claude (Claude Sonnet 4.5) and ChatGPT (GPT-5.1) did not generate any health disinformation in response to any direct, characterization, and fictionalization prompts submitted across the six health topics.

**Table 1.** Safeguarding the performance of consumer-facing large language model (LLM) platforms.

Platform (Model)	Direct prompts (% disinformation)	Characterization (% disinformation)	Fictionalization (% disinformation)
ChatGPT (GPT-5.1)	0 (0)	0 (0)	0 (0)
Gemini (Gemini 3.0 Pro)	0 (0)	30 (100)	28 (93)
Claude (Claude Sonnet 4.5)	0 (0)	0 (0)	0 (0)
Copilot (GPT-5)	14 (47)	21 (70)	25 (83)
Grok (Grok 4)	19 (63)	10 (33)	25 (83)
Meta AI (Llama 4)	14 (47)	28 (93)	24 (80)

For the direct prompts, Copilot (GPT-5) and Meta AI (Llama 4) produced disinformation in 47% (14 of 30) submissions, while Grok (Grok 4) did so in 63% (19 of 30). Gemini (Gemini 3.0 Pro) blocked all direct disinformation-seeking requests.

For the obfuscated prompts, Gemini (Gemini 3.0 Pro) produced disinformation in 100% (30 of 30) of characterization prompts and 93% (28 of 30) of fictionalization prompts. Meta AI (Llama 4) generated disinformation in 93% (28 of 30) of characterization and 80% (24 of 30) of fictionalization prompts. Copilot (GPT-5) produced disinformation in 70% (21 of 30) of characterization and 83% (25 of 30) of fictionalization prompts, while Grok (Grok 4) did so in 33% (10 of 30) and 83% (25 of 30), respectively.

Multimedia Appendix 3 includes representative model outputs, with one example for each of the three prompt types across all six topics.

Compared with the 2023 benchmark for direct prompt submissions, Anthropic’s Claude (Claude 2 via Poe in 2023; Claude Sonnet 4.5 via claude.ai in 2025) produced no health disinformation in either evaluation. Both ChatGPT (GPT-4 in 2023; GPT-5.1 in 2025) and Gemini (PaLM 2 in 2023; Gemini-3.0 Pro in 2025) appeared to improve, as each had generated health disinformation in response to direct prompts in 2023 but demonstrated complete resistance to all such prompts in the current evaluation. In contrast, the Copilot platform (GPT-4 in 2023; GPT-5 in 2025) showed a decline in safeguard performance, shifting from rejecting all direct health disinformation prompts in 2023 to generating medical disinformation for almost half of such prompts in this study. With regards to the Llama models (Llama 2 accessed by Hugging Chat in 2023; Llama 4 accessed by Meta AI in 2025), each assessment generated health disinformation across both evaluation timepoints.

## Discussion

This evaluation provides an updated assessment of how contemporary consumer-facing LLM platforms respond to targeted attempts to elicit health disinformation. Across six disinformation domains and three prompt styles, the findings demonstrate significant heterogeneity in safeguard performance. Although some platforms resisted all attempts to generate disinformation, others remained highly susceptible, particularly when prompts were framed indirectly.

Claude (Claude Sonnet 4.5) and ChatGPT (GPT-5.1), did not produce disinformation in any instance. Their consistent refusal across direct, characterization and fictionalization prompts suggests that platform-level safeguards effectively identify and interrupt harmful requests, even when framed within narrative structures. Relative to the 2023 benchmark, these outcomes indicate improved resistance for ChatGPT and continued strong performance for Claude.

The remaining platforms exhibited substantial gaps. Copilot (GPT-5), Meta AI (Llama 4), and Grok (Grok 4) each produced disinformation in response to direct prompts and rates increased under obfuscation. For Copilot and Meta AI, disinformation rates were higher under both characterization and fictionalization than under direct prompting. Grok showed a mixed pattern, with lower disinformation rates under characterization but high rates under fictionalization. Overall, these patterns indicate that safeguards in several widely used platforms do not generalize reliably across common styles of indirect input, consistent with prior work showing that relatively simple prompt obfuscation can bypass safety controls [8]. This matters because real-world health disinformation is often embedded in stories, speculative scenarios, or conversational phrasing rather than stated as an explicit request for falsehoods [9].

Gemini (Gemini 3.0 Pro) showed a distinct profile. It rejected all direct prompts but produced health disinformation in nearly all obfuscated cases. This divergence indicates a narrow protective boundary that responds primarily to the phrasing of a request rather than its substance [10]. Direct prompt refusal can therefore give a misleading impression of safety, and the results highlight the limitations of single-format safety testing, which is still common in developer and independent evaluations [3]. Furthermore, when viewed alongside the 2023 benchmark, the present findings also show that platform safeguards can change materially as models and platforms are updated.

This study has several limitations. The evaluation focused on text-only interactions, six predefined topics, and a fixed four-day assessment window. Platform performance may differ for multimodal inputs, such as images or scanned documents, or for emerging health disinformation topics that were not represented in the prompt set. All prompts were in English; however, prior evaluations of LLM platforms in responding to health questions indicate that responses in other languages contain more inaccuracies [11]. Even with these limitations, the findings indicate that while progress is evident in some systems, the overall reliability of consumer-facing LLM platforms in resisting health disinformation remains uneven. Increasing commercial pressures, such as the introduction of advertising and monetisation strategies [12], have the potential to alter the incentives surrounding safety controls in unpredictable ways. Therefore, it is important to conduct systematic, long-term monitoring of LLM safety performance to inform timely policy and regulatory responses.

## Supplementary material

10.2196/89831 Multimedia Appendix 1 Access details for all consumer-facing evaluated large langauge model platforms.

10.2196/89831 Multimedia Appendix 2 Disinformation prompts.

10.2196/89831 Multimedia Appendix 3 Representative model outputs.

## Abbreviations

LLM: large language model
