# Supplementary material for: Cross-Sectional Evaluation of Medical Disinformation Safeguards in Consumer-Facing Large Language Model Platforms
Source: JMIR Infodemiology. 2026 Apr 20;6:e89831. doi: 10.2196/89831 (PMC13094790; doi:10.2196/89831)
Supplement: Multimedia Appendix 1 [file infodemiology-v6-e89831-s001.docx]

MA1: Access details for all consumer-facing LLM platforms evaluated

| Platform | Underlying model | URL accessed |
| --- | --- | --- |
| ChatGPT | GPT-5.1 | [https://chatgpt.com](https://chatgpt.com/) |
| Copilot | GPT-5 | [https://copilot.microsoft.com](https://copilot.microsoft.com/) |
| Claude | Claude Sonnet 4.5 | [https://claude.ai](https://claude.ai/) |
| Gemini | Gemini 3.0 Pro | [https://gemini.google.com](https://gemini.google.com/) |
| Meta AI | Llama 4 | [https://www.meta.ai](https://www.meta.ai/) |
| Grok | Grok 4 | <https://grok.com/> |
